# The genome sequence of the colonial chordate, *Botryllus schlosseri*

**DOI:** 10.7554/eLife.00569

**Published:** 2013-07-02

**Authors:** Ayelet Voskoboynik, Norma F Neff, Debashis Sahoo, Aaron M Newman, Dmitry Pushkarev, Winston Koh, Benedetto Passarelli, H Christina Fan, Gary L Mantalas, Karla J Palmeri, Katherine J Ishizuka, Carmela Gissi, Francesca Griggio, Rachel Ben-Shlomo, Daniel M Corey, Lolita Penland, Richard A White, Irving L Weissman, Stephen R Quake

**Affiliations:** Department of Pathology, Institute for Stem Cell Biology and Regenerative Medicine, Stanford University, Stanford, United States; Hopkins Marine Station, Stanford University, Pacific Grove, United States; Departments of Applied Physics and Bioengineering, Howard Hughes Medical Institute, Stanford University, Stanford, United States; Dipartimento di Bioscienze, Università degli Studi di Milano, Milano, Italy; Department of Biology, University of Haifa-Oranim, Tivon, Israel; Ludwig Center for Cancer Stem Cell Research and Medicine, Stanford University School of Medicine, Stanford, United States; California Institute of Technology, United States

**Keywords:** Botryllus schlosseri, tunicates, stem cell, hematopoiesis, vertebrate evolution, genome, Other

## Abstract

*Botryllus schlosseri* is a colonial urochordate that follows the chordate plan of development following sexual reproduction, but invokes a stem cell-mediated budding program during subsequent rounds of asexual reproduction. As urochordates are considered to be the closest living invertebrate relatives of vertebrates, they are ideal subjects for whole genome sequence analyses. Using a novel method for high-throughput sequencing of eukaryotic genomes, we sequenced and assembled 580 Mbp of the *B. schlosseri* genome. The genome assembly is comprised of nearly 14,000 intron-containing predicted genes, and 13,500 intron-less predicted genes, 40% of which could be confidently parceled into 13 (of 16 haploid) chromosomes. A comparison of homologous genes between *B. schlosseri* and other diverse taxonomic groups revealed genomic events underlying the evolution of vertebrates and lymphoid-mediated immunity. The *B. schlosseri* genome is a community resource for studying alternative modes of reproduction, natural transplantation reactions, and stem cell-mediated regeneration.

**DOI:** http://dx.doi.org/10.7554/eLife.00569.001

## eLife Digest

The tunicates are an evolutionary group that includes species such as sea squirts and sea tulips. Their name comes from the structure known as a ‘tunic’ that surrounds their sac-like bodies. As marine filter feeders, tunicates obtain nutrients by straining food particles from water, and they can live either alone or in colonies depending on the species. Charles Darwin suggested that tunicates may be the key to understanding the evolution of vertebrates, and indeed today they are regarded as the closest living relatives of this group.

Colonial tunicates can reproduce either sexually, or asexually by budding. Compatible colonies have the ability to recognize one another and to fuse their blood vessels to form a single organism, whereas incompatible colonies reject one another and remain separate. This recognition process bears some resemblance to the rejection of foreign organ transplants in mammals.

Here, Voskoboynik and co-workers present the first genome sequence of a colonial tunicate, *Botryllus schlosseri.* They used a novel sequencing approach that significantly increased the length of a DNA molecule that can be determined by next-generation sequencing, and allowed large DNA repeat regions to be easily resolved. In total, they sequenced 580 million base pairs of DNA, which they estimate contains roughly 27,000 genes.

By comparing the *B. schlosseri* genome with those of a number of vertebrates, Voskoboynik et al. identified multiple *B. schlosseri* genes that also participate in the development and functioning of the vertebrate eye, heart, and auditory system, as well as others that may have contributed to the evolution of the immune system and of blood cells. The genome of *B. schlosseri* thus provides an important new tool for studying the genetic basis of the evolution of vertebrates.

**DOI:** http://dx.doi.org/10.7554/eLife.00569.002

## Introduction

In 1866, Russian embryologist Alexander Kowalevsky wrote to Charles Darwin about the extensive developmental and morphological similarities between ascidian larvae and vertebrates, leading Darwin to hypothesize that ascidians (belonging to urochordates or tunicates) might be crucial to understanding the origin of the vertebrate phylum (Darwin, 1874). Indeed, tunicates are the closest extant relatives of vertebrates (Delsuc et al., 2006), and represent an investigative model for evolutionary events leading to adaptive immunity (Sabbadin, 1962; Scofield et al., 1982) and vertebrate-specific organ/tissue complexity (Dehal et al., 2002; Jeffery et al., 2004; Abitua et al., 2012). The colonial tunicate species, *Botryllus schlosseri*, represents an important model organism for studying unique aspects of a pre-vertebrate colonial lifestyle, such as self recognition (Sabbadin, 1962; Scofield et al., 1982), vasculature and blood development (Schlumpberger et al., 1984; Gasparini et al., 2008; Tiozzo et al., 2008), apoptosis (Lauzon et al., 1993; Cima et al., 2009), and alternative reproduction pathways (Sabbadin et al., 1975; Manni and Burighel, 2006; Voskoboynik et al., 2007; Lemaire, 2011), including stem cell-mediated regeneration of complete individuals within a colony unit (Laird et al., 2005; Voskoboynik et al., 2008; Rinkevich et al., 2013).

**Figure 1.**
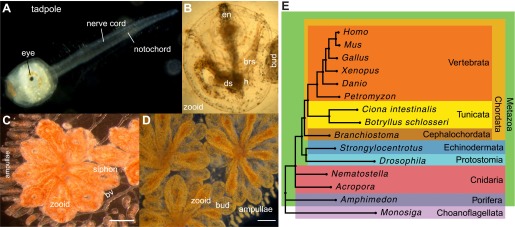
*Botryllus schlosseri* anatomy, life cycle, and phylogeny. *B. schlosseri* reproduces both through sexual and asexual (budding) pathways, giving rise to virtually identical adult body plans. Upon settlement, the tadpole phase of the *B. schlosseri* lifecycle (**A**) will metamorphose into a founder individual (oozooid) (**B**), which through asexual budding, generates a colony. The colony includes three overlapping generations: an adult zooid, a primary bud, and a secondary bud, all of which are connected via a vascular network (bv) embedded within a gelatinous matrix (termed tunic). The common vasculature terminates in finger-like protrusions (termed ampullae; **B**–**D**). Bud development commences in stage A (**C**). Through budding, *B. schlosseri* generates its entire body, including digestive (ds) and respiratory (brs) systems, a simple tube-like heart (h), an endostyle (en) that harbors a stem cell niche, a primitive neural complex, and siphons used for feeding, waste, and releasing larvae (**B**–**D**). Each week, successive buds grow large (**D**) and complete replication of all zooids in the colony, ultimately replacing the previous generation’s zooids, which die through a massive apoptosis. (**E**) A phylogenomic tree produced from analysis of 521 nuclear genes (40,798 aligned amino acids) from 15 species, including *B. schlosseri*. Scale bar-1 mm. **DOI:** http://dx.doi.org/10.7554/eLife.00569.003

*Botryllus schlosseri* is an invasive colonial urochordate, living in large communities consisting of multiple colonies organized into expansive mats that coat a variety of marine surfaces, such as rocks, molluscs, multicellular algae, and ship hulls (Stoner et al., 2002). Communities develop among compatible colonies, governed by a genetically encoded histocompatibility system (Sabbadin, 1962; Scofield et al., 1982). The progeny of each colony usually represents a clone of the vascularly connected, asexually reproducing individuals (zooids) derived from a single planktonic larva (Manni and Burighel, 2006; Figure 1A–D). Compatible colonies fuse their blood vessels to generate a chimera, while incompatible colonies reject one another, maintaining individuality (Sabbadin, 1962; Scofield et al., 1982; Voskoboynik et al., 2013). Following the fusion of blood vessels between colonies, the circulating stem cells of one partner colony can compete and replace the germline and/or the soma of the other partner (Stoner and Weissman, 1996; Stoner et al., 1999; Laird et al., 2005; Voskoboynik et al., 2008; Rinkevich et al., 2013), a phenomenon analogous to allogeneic transplantation.

**Figure 1—figure supplement 1.**
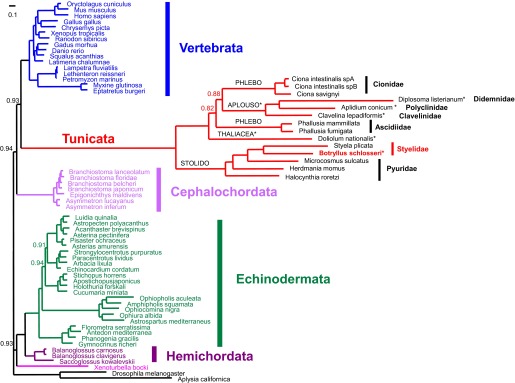
Mitogenomic analysis of tunicates and deuterostomes. Based on the 13 mitochondrially-encoded proteins. The tree was inferred by PhyloBayes under a GTR+G+CAT model. Support values at nodes represents Bayesian Posterior Probability (PP) and are reported only when >0.5 and <0.95. Nodes with PP < 0.5 were collapsed. The tree was rooted with the non-deuterostome *Drosophila* and *Aplysia* species. The main deuterostome lineages are represented in different colours. Abbreviations for tunicate orders: Stolido: Stolidobranchia; Phlebo: Phlebobranchia; Aplouso: Aplousobranchia. Colonial tunicates are indicated by an asterisk and include *Botryllus schlosseri*, all Aplousobranchia ascidians, and the thaliacean *Doliolum nationalis*. **DOI:** http://dx.doi.org/10.7554/eLife.00569.004

Tunicates are classified as chordates because their planktonic larva stage (Figure 1A) shares structural characteristics with all chordates: a notochord, dorsal neural tube, segmented musculature, and gill slits (Darwin, 1874; Dehal et al., 2002). Larvae settle in response to light and metamorphose into sessile individuals (Figure 1B), which lose most of their chordate phenotypes (Darwin, 1874; Dehal et al., 2002). Tunicates reproduce either sexually (solitary tunicates; Dehal et al., 2002; Lemaire, 2011), or sexually and asexually (colonial tunicates; Manni and Burighel, 2006; Lemaire, 2011). These two reproductive modes give rise to nearly identical complex adult body plans, including digestive and respiratory systems, a simple tube-like heart, siphons, an endostyle, a neural complex, ovary and testis (Manni and Burighel, 2006; Figure 1A–D).

The ability to reproduce asexually renders colonial tunicates robust survivors, capable of rapid proliferation and whole body regeneration. These unique features of colonial tunicates coupled with their key evolutionary position and long history of scientific study prompted us to sequence the *B. schlosseri* genome.

## Results and discussion

### A novel genome sequencing method for deciphering repeat-rich genomes

**Figure 2.**
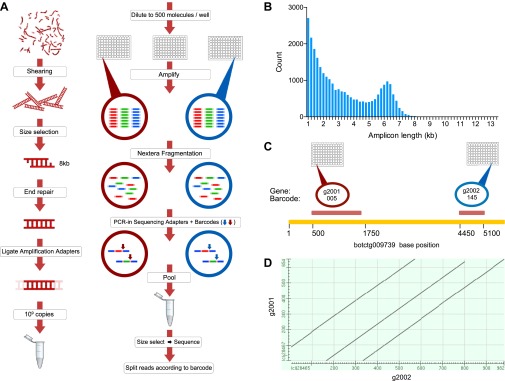
A novel short read genome sequencing and assembly method for complex, repeat-rich genomes. (**A**) Genomic DNA is sheared into 6–8 kb fragments, partitioned into twelve 96-well plates, further fragmented to 600–800 bp, barcoded and sequenced separately for each well (Illumina HiSeq 2000 2x100bp), and assembled by Velvet. (**B**) Size distribution of contigs assembled from a representative library preparation (BL5). (**C**) Limiting the number of amplifiable molecules per well (barcode) to the level that almost 100% of all amplifiable molecules are present as single copies (<1000 gDNA molecules) greatly reduces the chance of having a repeated or homologous sequence within a well. Thus, sample complexity is significantly reduced, which reduces ambiguity in the reconstruction of a consensus sequence. As an example, two different predicted repeat-containing genes (g2001,1189bp; and g2002, 688bp) were assembled from two different wells (005 and 145 respectively). Although they contain highly homologous repeats (represented as a Dot Matrix plot, (**D**) these repetitive genes were resolved and reconstructed properly in the final assembly. **DOI:** http://dx.doi.org/10.7554/eLife.00569.005

The *B. schlosseri* genome was previously estimated to be 725 Mb based on flow cytometry analysis (De Tomaso et al., 1998), and metaphase spreads suggested that it is organized into 16 chromosomes (Colombera, 1963). To accurately assemble this relatively large genome, we developed a novel method to accurately sequence many large fragments in parallel. This long read sequencing approach (LRseq) effectively increases the read length of a next generation sequencer by 50-fold, while decreasing the error rate by orders of magnitude (Figure 2; ‘Materials and methods’ under ‘Genome sequencing and assembly’). Our approach began with genomic DNA sheared to 6–8 kb fragments. Limiting dilution was used to create aliquots of a few hundred to a few thousand DNA molecules. Each aliquot was amplified with PCR, fragmented (600–800 bp), barcoded, and sequenced by Illumina HiSeq 2000 (Figure 2). The Velvet assembler (Zerbino, 2010) was used to assemble short paired-end reads from each barcode (i.e., well) separately, thus simplifying the assembly problem and creating effective read lengths corresponding to the original large fragment sizes (Figure 2B; Supplementary file 2A, Supplementary file 2B). Limiting the number of DNA molecules per well greatly reduces or eliminates chances of having a repeated or duplicate sequence within a defined partition. Furthermore, since each well was over-sequenced, the error rate is reduced by the coverage and is substantially improved from the intrinsic error rate of the sequencer (Supplementary file 2C). This procedure is amenable to automation in multiwell plates, and we obtained data from twelve 96-well plates (Supplementary file 2A, Supplementary file 2B). We validated this method on human genomic DNA, for which an independent reference is available (Figure 2—figure supplement 1).

**Figure 2—figure supplement 1.**
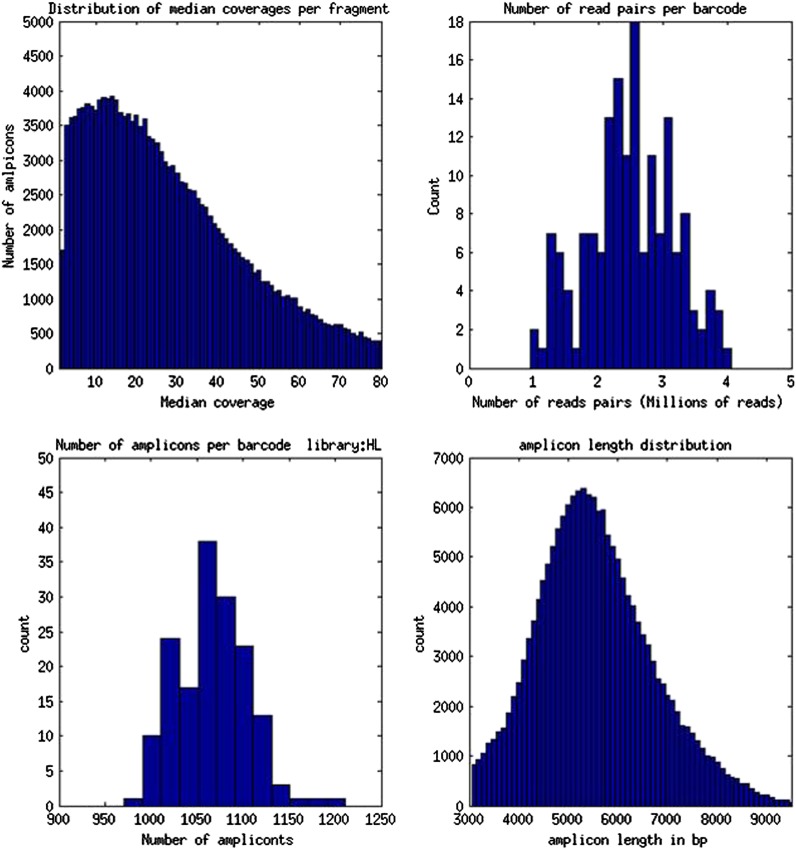
Validation of LRseq approach on human genomic DNA. Genomic DNA from HapMap NA7019 was prepared for LRseq. These figures show LRseq assembly statistics, obtained by mapping sequenced reads to human genome reference 36. These data were also used to estimate the concentration of amplifiable molecules in *B. schlosseri* 356a DNA samples prepared by an identical protocol. **DOI:** http://dx.doi.org/10.7554/eLife.00569.006

**Figure 2—figure supplement 2.**
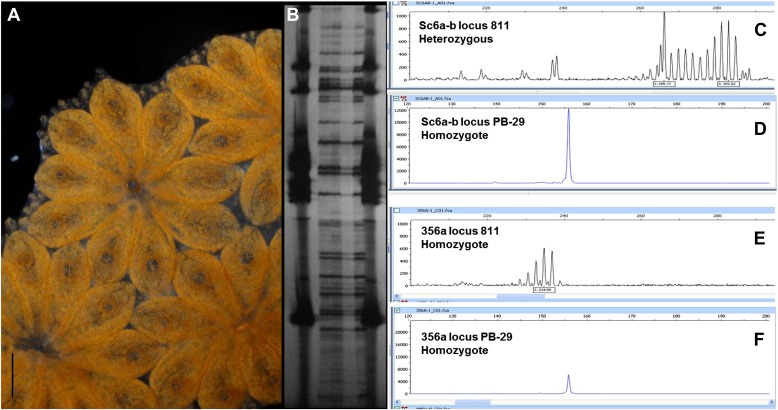
Clonality confirmation of the genome of clone Sc6a-b and clone 356a. (**A**) Sc6a-b clone, a long lived (7 years old when sampled), highly regenerative colony was chosen to be sequenced. Sc6a-b subclones were starved for 48 hr prior to sampling, and 400 individuals (zooids) were sampled for sequencing. Subclones of this colony are still alive and maintained in our mariculture facility. (**B**) A few zooids were taken from every sample set and tested via AFLP’s genotyping analysis, confirming that all zooids belong to one genotype. (**C** and **D)**. Sc6a-b microsatellite loci were homozygous (2 loci) and heterozygous (1 loci) confirming one genotype. (**E** and **F**) 356a clone was a highly regenerative long lived colony. 150 individuals were sampled and their gDNA was sequenced. Microsatellite loci were homozygous (**E** and **F**), confirming one genotype. Scale bar-1 mm **DOI:** http://dx.doi.org/10.7554/eLife.00569.007

**Figure 2—figure supplement 3.**
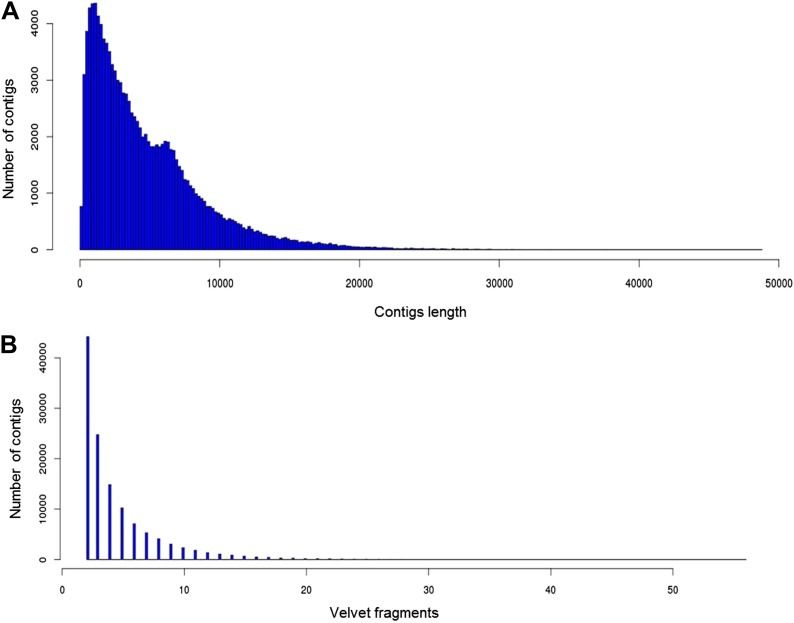
Statistics for 356a assembly. (**A**) Contig length distribution. (**B**) Distribution of coverage of 356a assembled Celera contigs by Velvet assembled fragments. **DOI:** http://dx.doi.org/10.7554/eLife.00569.008

**Figure 2—figure supplement 4.**
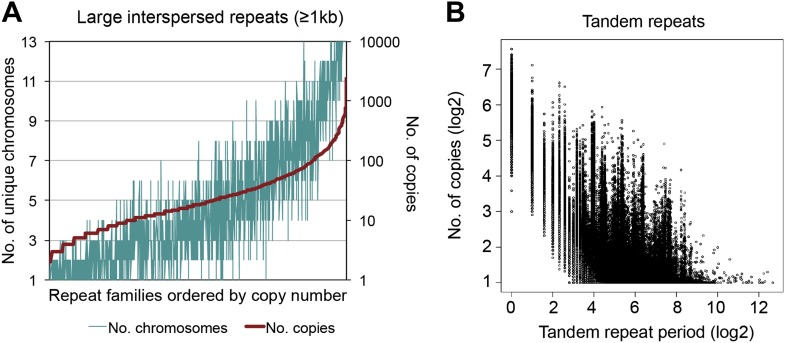
Interspersed and tandem repeats distribution in the *B. schlosseri* genome. (**A**) RepeatScout (version 1.0.5; Price et al., 2005) was used to identify interspersed repeat elements de novo using a k-mer length of 14. All identified repeats were subsequently filtered for tandem repeat and low complexity content, using RepeatScout. Genome-wide interspersed repeats were catalogued using RepeatMasker (version open-4.0; Smit et al., 1996-2010). The distribution of large interspersed repeats families (≥1kb) ordered by copy number is presented. (**B**) To identify both perfect (100% sequence identity) and degenerate genomic tandem repeats, we used XSTREAM (Newman and Cooper, 2007), with a minimum repeat length of 20 bp, minimum word match of 0.8, and otherwise default parameters. 3,183,988 tandem repeats were identified, period range: 1–6525 bp, copy number range: 2.7–1096x **DOI:** http://dx.doi.org/10.7554/eLife.00569.009

**Figure 2—figure supplement 5.**
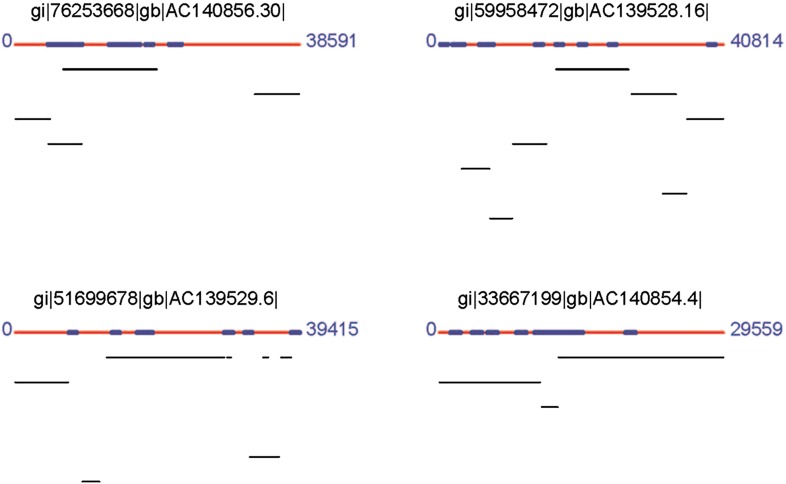
Coverage of 4 fosmids by the *B. schlosseri* assembly. Fosmid sequences (red lines; gi; ac numbers are shown, number=bp), were compared with *B. schlosseri* contigs using blast (e-value < e^−10^). Best alignments between contigs >500bp (black lines) are shown. Repetitive regions are marked (blue). **DOI:** http://dx.doi.org/10.7554/eLife.00569.010

**Figure 2—figure supplement 6.**
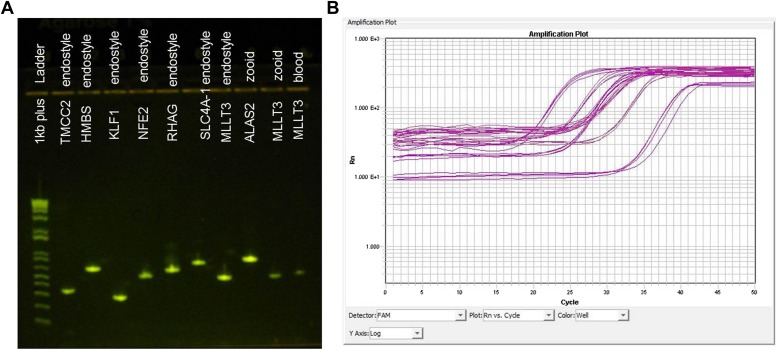
Validation of putative *B. schlosseri* genes. We experimentally validated 145 *B. schlosseri* predicted genes. Genes were validated by observing expression in *B. schlosseri* cDNAs and gDNA via PCR and qPCR assays and resequencing them on Sanger. (**A**) cDNA PCR product of several early erythroid and HSC putative genes identified in *B. schlosseri* tissues (endostyle, blood or zooid). Names of the putative genes and the tissues that were tested in this experiment are indicated on the gel image. (**B**) qPCR expression in *B. schlosseri* blood of six putative immunity genes. **DOI:** http://dx.doi.org/10.7554/eLife.00569.011

Genomic DNA (gDNA) was extracted from tissue from two long-lived *B. schlosseri* colonies (Sc6a-b and 356a) raised in our mariculture facility (‘Materials and methods’ under ‘Animals and genomic DNA sample collection’). Microsatellite heterogeneity confirmed clonality (Figure 2—figure supplement 2). Each colony was sequenced and assembled separately. We first attempted conventional sequencing and assembly from colony Sc6a-b DNA using Roche 454 Titanium (Branford, USA) and Illumina GAII (San Diego, USA) sequences (Supplementary file 2C, ‘Materials and methods’ under ‘Genome sequencing and assembly’). This Sc6a-b assembly achieved an average N50 of 1 kb, yielding short contigs that were insufficient for whole genome assembly (Supplementary file 2D). By contrast, when we applied LRseq to the 356a clone, we obtained a 566 Mbp assembly with a dramatically improved N50 of 7kb (Supplementary file 2D; Figure 2—figure supplement 3). This approach not only simplified the assembly of a complex eukaryotic genome, but also reduced the confounding impact of repetitive DNA on contig assembly (Figure 2C–D; Figure 2—figure supplement 3).

### Chromosome assignments, repeats, and gene content

**Figure 3.**
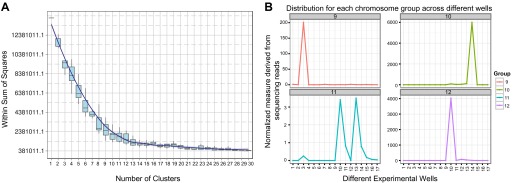
Clustering and assignment of *B.schlosseri* chromosomes. (**A**) We isolated and sequenced 21 metaphase chromosome mixtures using a microfluidic device. Each chromosome mixtures was amplified, barcoded and sequenced separately (IlluminaHiSeq). Genomic contigs larger than 7 kb were aligned to the chromosome reads using BWA. Subsequently, assignment of scaffolds to chromosome cluster was performed using iterative K-means clustering on the correlation matrix between each scaffold. In addition, to find the number of clusters/chromosomes we performed k-means clustering iteratively across different cluster numbers. This plot demonstrates that increasing beyond 13 clusters does little to reduce the error; therefore 13 chromosomes were successfully resolved. (**B**) To estimate the configuration after the clustering step, 17 out of the 21 wells were deduced to contain information that is used in the clustering process. The average number of normalized reads counts from each metaphase chromosome mixture (well) that align to each scaffold in a cluster group was calculated and plotted. Each peak represented can be inferred to denote the presence of a specific chromosome in the well. Examples of four representative wells are presented, metaphase chromosome mixtures contained between 1–4 chromosomes (see also Figure 3—figure supplement 1). **DOI:** http://dx.doi.org/10.7554/eLife.00569.012

We sought to determine the chromosomal organization of the *B. schlosseri* genome. Using embryos from a wild *B. schlosseri* colony from Monterey Bay, we loaded a dilute solution of dispersed metaphase chromosomes into a microfluidic device as previously described (Fan et al., 2011). The isolated metaphase chromosome mixtures from 21 individual wells were amplified, barcoded, and sequenced separately (‘Materials and methods’ under ‘Chromosome sequencing, assignment and assembly’; Fan et al., 2011; Xu et al., 2011). Using the 21 chromosome mixtures, containing between 1 and 4 chromosomes each, 356a genomic contigs larger than 7 kb were aligned to the chromosome reads using BWA. Then, scaffolds were assigned to chromosome clusters by iterative K-means clustering on the correlation matrix between each scaffold (Figure 3; ‘Materials and methods’ under ‘Chromosome sequencing, assignment and assembly’). Assuming that *B. schlosseri* carries 16x2 chromosomes (Colombera, 1963), this approach clearly resolves 13 chromosomes with a mean chromosome meta-scaffold size of 16,234 kb and a mean N50 of 38 kb (Figure 3; Figure 3—figure supplement 1; Supplementary file 2D). Finally, we attempted to improve our genomic assembly by incorporating the additional 21 chromosome assemblies into a hybrid assembly (‘Materials and methods’ under ‘Chromosome sequencing, assignment and assembly’; Figure 3—figure supplement 2; Figure 3—figure supplement 1). An overall improvement in N50 was achieved, yielding a final 580 Mbp draft assembly (Supplementary file 2D).

**Figure 3—figure supplement 1.**
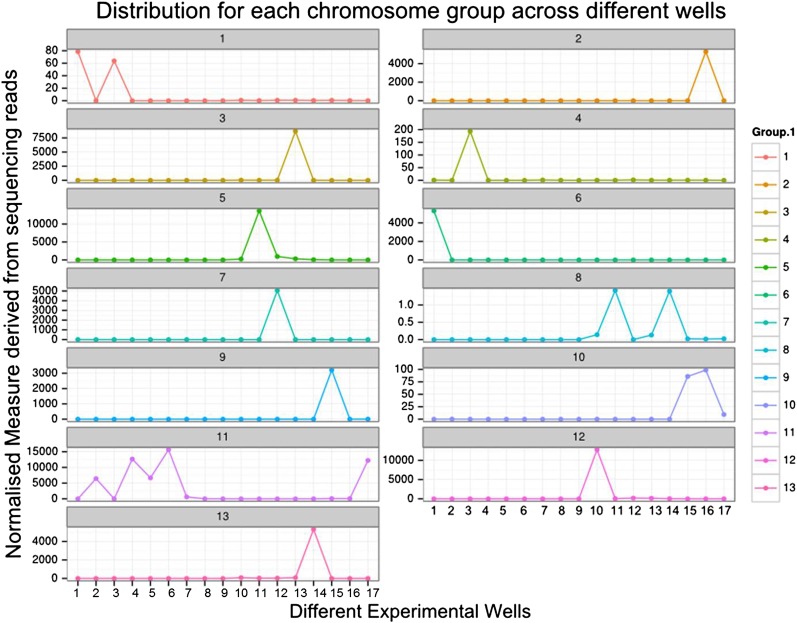
Distribution of *B. schlosseri* chromosome groups across different wells. We isolated and sequenced metaphase diluted chromosome mixtures using a microfluidic device. Each chromosome mixture was amplified, barcoded and sequenced separately (IlluminaHiSeq). The average number of normalized reads counts from each diluted chromosome mixture (well) that align to each scaffold in a cluster group was calculated and plotted. Each peak represents the presence of a specific chromosome in the well. In the 17 wells presented above, chromosome mixtures contained between 1–4 chromosomes. **DOI:** http://dx.doi.org/10.7554/eLife.00569.013

**Figure 3—figure supplement 2.**
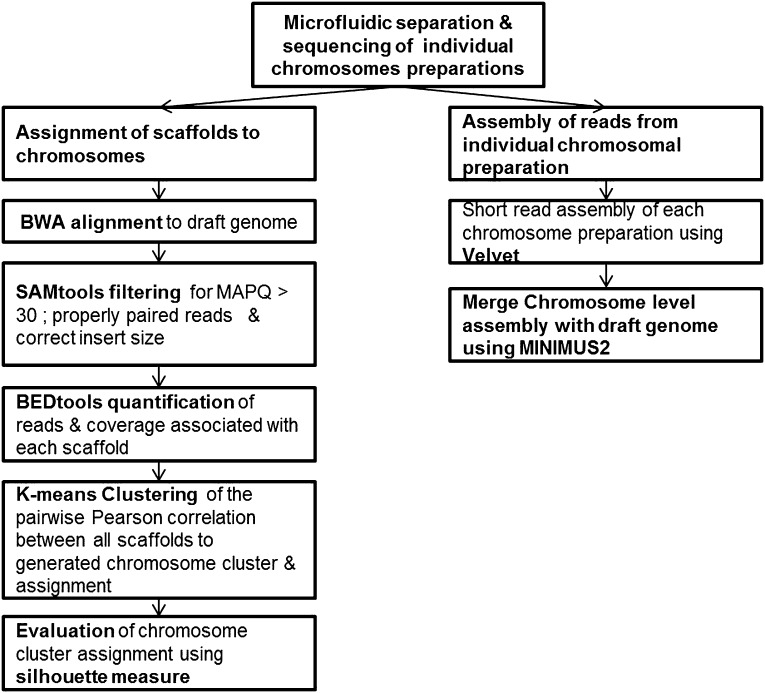
Pipeline for the assignment of chromosome scaffolds and the 356a–chromosomes hybrid assembly process. **DOI:** http://dx.doi.org/10.7554/eLife.00569.014

**Figure 3—figure supplement 3.**
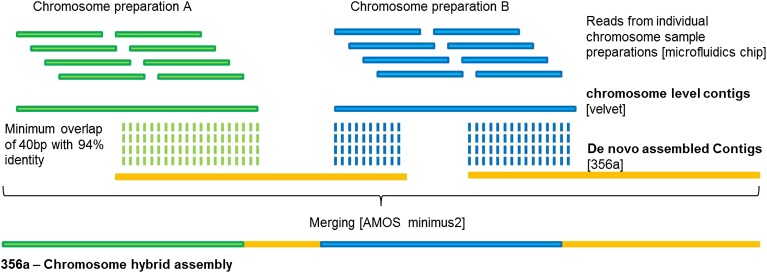
356a-Chromosome hybrid assembly of *B. schlosseri*. Reads from each of the individual chromosome sample preparations were subsequently assembled using Velvet. The resulting chromosome level contigs were then merged with the 356a assembly to create a 356a-chromosome hybrid assembly. **DOI:** http://dx.doi.org/10.7554/eLife.00569.015

**Figure 3—figure supplement 4.**
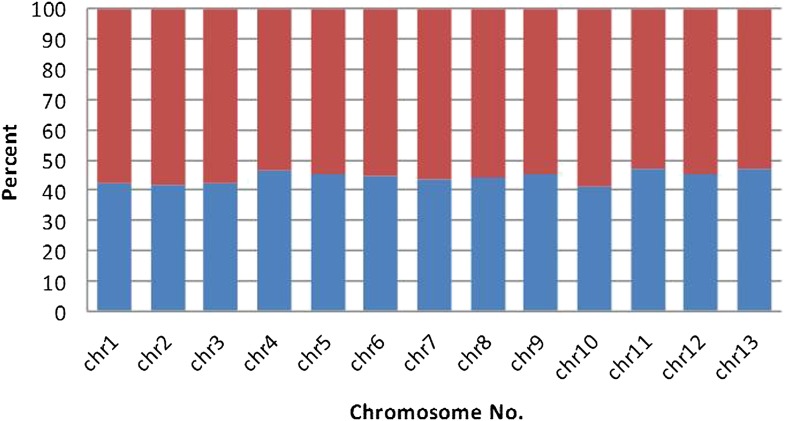
The fraction of *B. schlosseri* predicted intron-less genes (blue) and genes with introns (red) in the different chromosomes. **DOI:** http://dx.doi.org/10.7554/eLife.00569.016

Repetitive elements can confound traditional genome assembly methods (Salzberg et al., 2012), and are often removed to avoid assembly errors (e.g., Dehal et al., 2002; Putnam et al., 2007; Shinzato et al., 2011; Supplementary file 2H). However, because LRSeq was designed to explicitly resolve long read sequences even in the presence of repeats, we further evaluated LRSeq performance by enumerating two major repeat classes in the assembly, interspersed repeats and tandem repeats. We used RepeatScout for de novo identification of interspersed elements (Price et al., 2005), and RepeatMasker (Smit et al., 1996–2010) for analysis of genome-wide repeat demographics. We identified 6601 interspersed repeat families, each present in at least three copies, that together cover ∼65% of the *B. schlosseri* genome assembly (Supplementary file 2E). We also identified 1400 large repeat families, defined as interspersed repeats with genomic alignments of at least 1 kb. Notably, large interspersed repeats are found in a median of four chromosomes (of 13 chromosome assignments), and >10% of large interspersed repeat families occur in over 100 copies (Supplementary file 2E; Figure 2—figure supplement 4A). Despite considerable repetitive content, we observed a strong concordance between genomic contigs and Sanger fosmid sequences, supporting the effectiveness of the LRseq approach (e.g., see Figure 2—figure supplement 5). As a further validation, we interrogated our former sc6ab 380 Mb assembly for the same interspersed repeat elements, with the expectation of recovering less repeats. Indeed, only 52.27% of sc6ab base pairs were masked using the same repeat library. These results validate the repeat families and support their widespread presence in the *B. schlosseri* genome. Finally, we analyzed the assembly for perfect (100% sequence identity) and degenerate tandem repeat content using XSTREAM (Newman and Cooper, 2007). In all, ∼3.2 million tandem repeats were identified, with periods ranging from 1–6525 bp and copy numbers ranging from 2–1096x (Figure 2—figure supplement 4B). By comparison, the human genome was assembled to a very high standard using conventional Sanger technology and later Illumina technology, and was found to contain over 50% repeats (de Konning et al., 2011). The considerable repeat content and diversity in the *B. schlosseri* genome indicates that LRseq may have general utility for resolving repeat architectures in diverse eukaryotic genomes.

We further validated the assembly by comparison to a variety of independently generated *B. schlosseri* sequence data. All *B. schlosseri* genes (n = 66), fosmid sequences (n = 11) and most of the 98,611 expressed sequence tags (ESTs) available from NCBI aligned with the *B. schlosseri* draft assembly (Supplementary file 2F, Figure 2—figure supplement 5; ‘Materials and methods’ under ‘Evaluation of 365a-chromosomes hybrid assembly’). Moreover, nearly all of the independently sequenced and assembled Roche 454 Sc6a-b contigs (93%) were successfully mapped to the assembly (Supplementary files 2F; ‘Materials and methods’ under ‘Evaluation of 365a-chromosomes hybrid assembly’). Taken together, these data represent independent validation of the quality and integrity of the *B. schlosseri* draft assembly which compares favorably to, and in some cases exceeds, existing wild type genomes with respect to ungapped chromosome contig N50, chromosome assignments, and repeat sequence integration (e.g., see Supplementary file 2G).

Next, to identify protein-encoding genes, we generated RNA-Seq data (88 Gb; Supplementary file 2C) from 19 different colonies to guide the gene prediction program Augustus (Stanke et al., 2008). In total, 38,730 putative protein-coding loci were identified, all of which have at least 30% transcript support (‘Materials and methods’ under ‘RNA sequencing’, ‘Gene prediction’, ‘Gene annotation’; Supplementary file 2I). Among these predicted genes, 27,463 include a start and stop codon, 13,910 genes have at least one intron, and 13,553 are intron-less (Supplementary file 2H). Moreover, for each of the *B. schlosseri* chromosomes 55% of genes have at least one intron while ∼45% are intron-less (Figure 3—figure supplement 4). In addition, the mean *B. schlosseri* gene length is predicted to be 3.6 kbp with a mean exon length of 170 bp (Supplementary file 2H). We tested a set of 145 genes by PCR and Sanger-sequencing, and were able to confirm 144 of them (99.3%), further validating the genome assembly (Figure 2—figure supplement 6, ‘Materials and methods’ under ‘Evaluation of genes’).

Using these predicted genes, we investigated the evolutionary position of *B. schlosseri*. Phylogenomic analysis of 425 conserved homologous genes across 15 diverse species, and mitogenomic analysis of 65 species both support the phylogenic position of tunicates within Chordata (Delsuc et al., 2006; Figure 1E; Figure 1—figure supplement 1, Supplementary file 1; ‘Materials and methods’ under ‘Mitochondrial phylogeny’, ‘Phylogenomic analyses’), and provide strong evidence that colonial and solitary tunicates represent the closest living relative of vertebrates.

### *B. schlosseri* and the emergence of vertebrate phenotypes

We investigated the *B. schlosseri* genome for molecular events underlying the emergence and early diversification of vertebrates. Protein-encoding genes in *B. schlosseri* were compared to a diverse sampling of 18 well-annotated genomes from other species, and for each genome, we assessed the presence or absence of significant homology to human or mouse proteins (Figure 4—source data 1A; ‘Materials and methods’ under ‘Evolution analysis’). All proteomes were combined into a single data set (of constant size) for blast analysis. As such, differences in the number of genes per genome would not have impacted our results. An e-value cutoff of e^−10^ was selected to strike a balance between statistical significance and the detection of remote homology (‘Materials and methods’ under ‘Evolution analysis’). Among the analyzed species, we found that 77% of human genes could be traced back to protochordates with at least some homology (e-value ≤ e^−10^), around 10% less than chicken (85%) and frog (86%) genomes, indicating that the common ancestral genome of tunicates and vertebrates had homology to at least 77% of the human gene repertoire. This list includes about 660 genes present in the common ancestor, but absent in non-chordate species (Figure 4—source data 1B).

**Figure 4.**
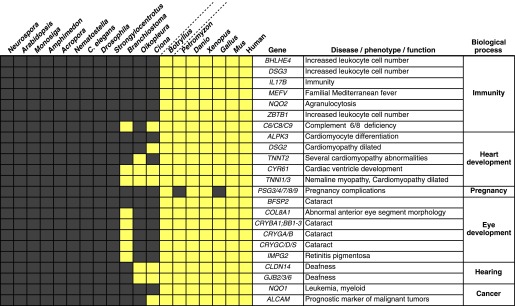
Innovations underlying the emergence and early diversification of vertebrates. Protein-encoding genes in *B. schlosseri* were compared to a diverse sampling of 18 well-annotated genomes from other species, and for each genome, the presence or absence of homology to human or mouse proteins was assessed (all vs all blastp e-value threshold of 1e^−10^; Figure 4—source data 1A). Our data indicate that homologs of ∼660 human/mouse genes were present in the common ancestor of tunicates and vertebrates, but not non-chordate species Figure 4—source data 1B). Among them are genes associated with the development, function, and pathology of vertebrate features, including heart, eye, hearing, immunity, pregnancy and cancer (Figure 4—source data 1C). Gray box = no homology; Yellow box = homology. **DOI:** http://dx.doi.org/10.7554/eLife.00569.017 10.7554/eLife.00569.018Figure 4—source data 1.Vertebrates evolution.(**A**) Innovations that underline the emergence and early diversification of vertebrates. We compared protein-encoding genes in *B. schlosseri* to a diverse sampling of 18 well-annotated genomes from other species. All protein sequences were compared by blastp against all other protein sequences. Based on this data set a list was generated of genes known from human and mouse and their existence (1) or absence (0) in the tested species (e-value < e^−10^). (**B**) The 660 putative genes present in protochordates, human and mouse, but absent in non-chordate species. This list was generated from Figure 4—source data 1A. Per every species, or species group we filtered for genes that were present in the tested species/species group and in human or mouse, but were absent in non-chordate species. (**C**) Innovations that underline the emergence and early diversification of vertebrates. This table is based on data gathered in Figure 4—source data 1B and is focused on the genes that are present in *B. schlosseri* and vertebrates (either alone or in combination with other protochordate species) but are absent in non-chordate species. A ToppGene analysis is presented of these sets of genes which summarized their molecular functions, biological processes, human and mouse phenotypes, and pathways they are involved in, gene families, drugs and human diseases.**DOI:** http://dx.doi.org/10.7554/eLife.00569.018

Among the genes found in *B. schlosseri* (either alone or in combination with other protochordate species) and vertebrates (Figure 4—source data 1B, Figure 4—source data 1C), we found genes that are critical to the development and function of the vertebrate heart (e.g., *ALPK3*, *TNNT2*; Hosoda et al., 2001; Frey et al., 2012), and eye (gamma and beta crystallins; Sun et al., 2011), and the ability to hear (*GJB*2/3/6 *CLDN*; Rabionet et al., 2000; Wilcox et al., 2001; Figure 4, Figure 4—source data 1C). Mutations in these genes are implicated in a variety of human diseases and disorders, including heart diseases (Frey et al., 2012), cataracts (Sun et al., 2011), deafness (Rabionet et al., 2000; Wilcox et al., 2001), and nemaline myopathy (Johnston et al., 2000; Figure 4, Figure 4—source data 1C). In addition, *B. schlosseri* was the only protochordate in our analysis with proteins homologous to pregnancy-specific glycoproteins (PSGs). PSGs are the major placental polypeptides, and complications in pregnancies and spontaneous abortions have been associated with abnormally low levels of PSGs in the maternal blood (Camolotto et al., 2010). Analogous to mammalian pregnancies, a common blood supply among kin is established and tolerated in *B. schlosseri* chimeras (Voskoboynik et al., 2009). Thus, by studying PSG-like proteins in *B. schlosseri*, new insights might be gained into maternal and fetal medicine.

Numerous genes predicted to have evolved in a common ancestor of *B. schlosseri* and vertebrates are essential to the immune system and hematopoiesis (Figure 4, Figure 4—source data 1B, Figure 4—source data 1C). Six genes unique to *B. schlosseri* and vertebrates (*ZBTB1*, *MEFV*, *DSG3*, *NQO1*, *NQO2* and *BHLHE40*) are associated with increased leukocyte and hematopoietic cell numbers (Figure 4—source data 1C; Chen et al., 2009). In our analysis, these genes are absent in cephalochordates and solitary urochordates, which all lack a defined vascular system (Moller and Philpott, 2005; Lemaire, 2011). In contrast, the heart in each individual zooid in a *B. schlosseri* colony beats synchronously with the hearts of other zooids in the colony, driving a bidirectional blood flow throughout an interconnected vasculature (Video 1). Moreover, this blood system carries at least ten morphologically different cell types (Schlumpberger et al., 1984; Ballarin et al., 2008). Because of the anatomy of *B. schlosseri*, coupled with its hematopoietic-related gene repertoire, we hypothesize that colonial ascidians may have retained and elaborated many components of the ancestral hematopoietic program, much of which has been lost in extant solitary urochordates and cephalochordates.

**Video 1.** *B. schlosseri* blood circulation. (**A)** Time-lapse acquisition of blood flow in the blood vessels (bv) and ampullae of a chimeric *B. schlosseri* colony, generated from a fusion between a mother and its offspring (fused). (**B**) Ampullae contract, buds develop, and a colony gets ready to replace the old generation. (**C**) Old generation zooids are getting resorbed (res. z) and replaced by the new generation (buds). (**D**) A heart beating and pumping blood in the primary bud of a different colony. (**E**) Blood flow through a common blood vessel between two allogeneic/compatible colonies, creating a natural chimera. **DOI:** http://dx.doi.org/10.7554/eLife.00569.019

### Evolution of hematopoiesis

**Figure 5.**
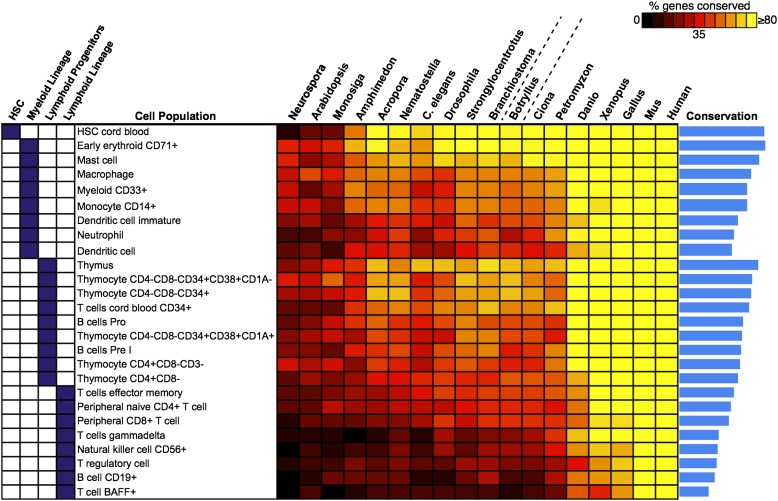
Analysis of blood and immune cell type-specific genes across evolution reveals evidence for hematopoietic precursors in *B. schlosseri*. We analyzed gene expression microarray data from 26 different human blood cell populations, organized into four cell lineages (HSC; Lymphoid Progenitors; Myeloid and Lymphoid Lineage), and identified a set of twenty signature genes with highly enriched expression profiles for each population (Supplementary file 3). For each blood-related gene set, we identified homologous gene sequences in *B. schlosseri* and 17 other species; the fraction of genes (out of 20) found for each species is displayed as a heat map. Within each major lineage, cell populations are sorted in decreasing order by a conservation index, calculated as the average number of genes found across the 18 species (indicated by a blue bar graph). **DOI:** http://dx.doi.org/10.7554/eLife.00569.020

We next attempted to identify potential precursors of human hematopoietic populations in *B. schlosseri* and 17 other diverse species, including fungal, plant, and mammalian species. We analyzed gene expression microarray data from 26 different human blood cell populations, and additional non-blood human tissue samples. We identified a set of twenty signature genes that were highly expressed in each of the 26 hematopoietic populations (Benita et al., 2010; Seita et al., 2012; ‘Materials and methods’ under ‘Evolution analysis’). For each blood-related gene set, we identified homologous gene sequences in *B. schlosseri* and 17 other species (Figure 5, Supplementary file 3). Among *B. schlosseri* homologs, we found high enrichment for gene sets predominantly expressed in human hematopoietic stem cells (HSCs; i.e., 14 of 20 cord blood HSC genes), myeloid populations (i.e., 14 of 20 early erythroid CD71+ genes), and early but not mature lymphoid populations. Consistent with previous studies (Bartl et al., 1994; Laird et al., 2000; Dishaw and Litman, 2009; Guo et al., 2009; Flajnik and Kasahara, 2010; Bajoghli et al., 2011; Hirano et al., 2011) this analysis indicates that the evolution of adaptive immunity progressed rapidly beginning with jawless vertebrates, with much of the genetic repertoire in place by the emergence of jawed vertebrates (Figure 5). However, homologs of human genes with specific expression in HSC and blood progenitor populations, including T and B progenitor cells, appear early in metazoan evolution (Figure 5; Supplementary file 3).

Unlike solitary tunicates (e.g., *Ciona*), *B. schlosseri* has a defined vasculature with circulating blood cells (including cells with lymphocyte-like and macrophage-like morphology; Schlumpberger et al., 1984; Ballarin et al., 2008; Video 1). As such, we further investigated by PCR and re-sequencing the expression of all 28 *B. schlosseri* genes with homology to human HSCs (n = 14) and early erythroid CD71+ blood cell (n = 14) gene sets. Strikingly, we found evidence for expression of 13 HSC homologs in the *B. schlosseri* endostyle stem cell niche (Voskoboynik et al., 2008), and 7 in the vasculature. We also confirmed expression of all 14 early erythroid CD71+ genes in the vasculature and endostyle (Supplementary file 3). Thus, our analysis not only identified *B. schlosseri* genes that may define evolutionary precursor cells of human hematopoietic lineages, but also indicates that the evolution of hematopoiesis proceeded from stem cells to myeloid populations to lymphoid populations, leading to the eventual emergence, absent in *B. schlosseri*, of T/B-cell based adaptive immunity in vertebrates (Figure 5; Supplementary file 3).

Not surprisingly, the *B. schlosseri* genome lacks significant homology to most genes known to play an important role in the vertebrate adaptive immune system. For instance, no evidence for the following immune-related genes could be found: (i) assembled major histocompatibility genes, (ii) genes with homology to *RAG1*/*RAG2*, which are involved in immunoglobulin and T-cell receptor rearrangements, (iii) terminal deoxynucleotidyl transferase, which adds nucleotides to the rearranging VDJ elements to create receptor diversity, (iv) V region subgenic elements encoding T cell and immunoglobulin antigen receptor domains, or (v) VLR like immune receptor elements found in lampreys (Weigert et al., 1970; Davis et al., 1984; Oettinger et al., 1990; Fagan and Weissman, 1998; Laird et al., 2000; Muramatsu et al., 2000; Pancer et al., 2004; Rogozin et al., 2007; Dishaw and Litman, 2009; Flajnik and Kasahara, 2010; Hirano et al., 2011). We identified a large fraction (∼45%; Supplementary file 2H; Figure 3—figure supplement 4) of intron-less genes in the *B. schlosseri* draft genome, including retroviral genes such as *Gag*, *Poli*, *Env* and LTRs, which are used by viruses to insert their genetic sequences into the host genomes. As adaptive immunity genes like *RAG1*/*RAG2* are intron-less and first appear in jawed vertebrates, it has been suggested that they may have originated via horizontal infections of primitive retroviral like agents, and/or gene transfer (Bartl et al., 1994). In addition, the *B. schlosseri* genome encodes homologues of *Foxn1*, the thymus epithelial gene mutated in the immunodeficient nude mouse (nu/nu), a marker of the thymopoietic microenvironment in vertebrates (Nehls et al., 1996; Bajoghli et al., 2011). These data indicate that at least some genetic circuitry relevant for vertebrate adaptive immunity was already in place in the common ancestor of the protochordate *B. schlosseri* and vertebrates. It leaves open the question of whether Ig or TCR genes, and the MHC proteins that capture and present intracellular peptides to T cells expressing these TCR proteins, existed in antecedents to *B. schlosseri* but were lost or somehow introduced after the line from colonial tunicates to the organisms that have an adaptive immune system. As *omnis DNA e DNA*, this question is perhaps the most puzzling of our findings.

In conclusion, using a novel method for deciphering eukaryotic genomes, we assembled and analyzed the *B. schlosseri* genome, the first colonial tunicate to be sequenced. One of the great challenges in evolutionary biology is to understand how differences in DNA sequences between species underlie distinct phenotypes. The *B. schlosseri* genome provides an important new resource for unraveling the genes and regulatory logic that led to the emergence of vertebrates and lymphoid-mediated immunity. Moreover, the many important features encoded by the *B. schlosseri* genome will facilitate new insights into complex vasculature, chimerism among kin, whole-body stem cell-mediated regeneration, and a colonial lifestyle.

## Materials and methods

### Animals and genomic DNA sample collection

Mature reproductive colonies of *Botryllus schlosseri* (Pallas) were collected from Santa Cruz and Monterey marinas, California . Hatched larvae were settled raised, and crossed in our mariculture facility as described (Boyd et al., 1986; De Tomaso et al., 1998). Long lived, highly regenerative colonies Sc6a-b and 356a, were chosen to be sequenced. Sc6a-b subclones were starved for 48 hr prior to sampling (to minimize DNA contamination), and 400 individuals (zooids) were sampled for gDNA sequencing. Subclones of this colony are still alive and maintained in our mariculture facility (Figure 2—figure supplement 2A). 150 individuals from colony 356a were sampled and their gDNA was sequenced. To confirm that all zooids belonged to one genotype, a few zooids were taken from every sample set and screened for polymorphism via amplified fragment length polymorphism (AFLP) analysis as described in (Voskoboynik et al., 2008) and microsatellite loci analysis as described in (Stoner et al., 2002), confirming one genotype for Sc6a-b and 356a colonies (Figure 2—figure supplement 2B–F). Tissue samples were dissected and flash-frozen in liquid nitrogen. Genomic DNA samples were extracted using a modified version of the Hoss and Paabo protocol (Hoss and Paabo, 1993) as described (De Tomaso et al., 1998).

### Genome sequencing and assembly

The *B. schlosseri* genome sequence assembly was performed using two independent methods.

**Colony Sc6a-b** genome sequence data was obtained using single read and paired-end protocols on the Roche (Roche, Branford, CT) 454 GS-FLX and Illumina Genome Analyzer II (GAII; Illumina, San Diego, CA) instruments (Supplementary file 2C). Sc6a-b gDNA was fragmented, libraries prepared, and sequencing conducted according to the manufacturer’s protocols. The 454 platform generated a total of 3086 Mb sequence data, the Illumina platform generated 3597 Mbp sequence data .The 6683 Mbp of sequence data obtained corresponds to ∼11-fold coverage of the *B. schlosseri* genome (estimated size of 600 Mbp, 454kmer estimation; Supplementary file 2C). The 454 shotgun and Illumina GAII paired-end reads were assembled de novo using Newbler v2.5 (Roche) with default settings, heterozygote mode. 380 Mbp comprised of 518,856 contigs were assembled with N50 of 1160 bp (Sc6a-b draft assembly; Supplementary file 2D).

**Colony 356a** We developed a novel method to obtain a sequence in order to assemble larger contigs and reduce assembly complexity. Colony 356a gDNA was sheared using HydroShear (speed setting 16; 20 cycles) into random fragments of 6–10 kb. Sheared gDNA was run on a 0.8% agarose gel, the 6–8 kb band was cut and the DNA extracted using Qiagen gel purification kit. Fragmented DNA was repaired using NEB end repair module (E6050S) to produce blunt ends. Blunt end DNA was purified on a Qiagen column. After purification standard double stranded adapters from the Roche 454 kit were ligated with NEB Quick Ligase, following Roche 454 Titanium protocols. 454 adaptor mix 27,145, a mix of two sequences was used:

Primer A1: 5′-CCATCTCATCCCTGCGTGTCTCCGACTCAG-3′; 3′-TCTCCGACTCAG-5′

Primer B: 5″-/5BioTEG/CCTATCCCCTGTGTGCCTTGGCAGTCTCAG-3′; 3′-TGGCAGTCTCAG-5′

Amplification primer mix: Ti forward: 5′-CCATCTCATCCCTGCGTGTC-3′; Ti reverse: 5′-CCTATCCCCTGTGTGCCTTG-3′

These adapters serve as priming sites for the downstream amplification of the long fragment library. Following DNA purification (Agencourt AMPureXP bead purification) and fill-in reaction, second size selection was performed to remove adapter dimers and narrow down DNA size range (6–8 kb). Qiagen gel purification kit was used to purify DNA. PCR amplification for long range amplification was performed as follows: initial denaturation at 94°C for 30 s, followed by 23 cycles of (94°C for 15 s, 65°C for 7 min), followed by a final extension 65°C for 7 min. Concentration of amplifiable molecules carrying both amplification adapters was estimated by comparing *B. schlosseri* samples to a human standard sample prepared using an identical protocol. qPCR with nonspecific intercalating dye (EvaGreen, Biotinum) was used to calculate concentrations. Human standard was prepared following an identical protocol from genomic DNA derived from HapMap sample NA7019. The amount of amplifiable DNA was obtained by mapping short reads to a human genome reference 36 and measuring the fraction of the genome that was covered (Figure 2—figure supplement 1). Mapping was done using Novoalign with default settings, counting the amount of bases covered by regions of more than 1500 bp with at least 2x coverage of properly mapping paired end reads. We then aliquoted the resulting library of *B. schlosseri* gDNA with amplification adapters into wells of two 96-well plates such that, on average, each well contained a predefined amount of amplifiable molecules (estimated number of 200–2000 molecule per well; 1–6 Mb of total amplifiable sequence). Randomly sampled molecules in each well were amplified using NEB LongAmp master mix in the presence of 400 nM of primers: LA-V2-LEFT 5′-CCATCTCATCCCTGCGTGTCTCCG-3′; LA-V2-RIGHT 5′-CCTATCCCCTGTGTGCCTTGGCAGT-3′) complementary to previously ligated adapters following the two-step protocol. The resulting library of amplicons was purified using Zymo ZR-96 DNA Clean & Concentrator-5 kit. Purified DNA was eluted into two 96-well plates according to the manufacturer’s protocol. DNA was fragmented and tagged using Nextera DNA sample prep kit. Following the standard protocol, samples were incubated for 5 min at 55°C in the provided high molecular weight buffer (Nextera DNA sample prep kit, Epicentre). Fragmented DNA was purified using Zymo ZR-96 DNA Clean & Concentrator-5 kit and was converted into Illumina compatible sequencing library using a custom protocol. Four oligos described in the Nextera DNA sample prep kit (Epicentre and Illumina) were added to the every well containing purified fragmented DNA in concentrations recommended by Epicentre.

Adaptor 1*: 5′AATGATACGGCGACCACCGAGATCTACACGCCTCCCTCGCGCCATCAG-3′; Primer 1*: 5′-AATGATACGGCGACCACCGA-3′; Primer 2*: 5′-CAAGCAGAAGACGGCATACGA-3′; Adaptor 2: 5′-CAAGCAGAAGACGGCATACGAGAT-[BAR CODE]*-CGGTCTGCCTTGCCAGCCCGCTCAG-3′

Adapter 2 carried a 7 bp barcode sequence unique for each well of two 96-well plates (Supplementary file 2I). Amplification was done using NEB Phusion GC master mix (2x) following the recommended Nextera limited cycle PCR protocol designed to incorporate barcoded adapters: 72°C for 3 min, 95°C for 40 s, followed by nine cycles of 62°C for 30 s, 72°C for 3 min. The resulting 192 Illumina libraries were pooled together and purified using Qiagen Quiavac96 DNA purification kit. Size selection was performed by running a 2% agarose gel and excising the 400–900 bp band. This gel band was purified (Qiagen), quantitated using Agilent Bioanalyzer 2100 High-Sensitivity chip and sequenced on Illumina HiSeq 2000 sequencer following manufacturer recommended protocols. After sequencing, multiplexed libraries reads were de-multiplexed and separated into independent files (according to barcodes). Reads were then screened to remove reads with low overall quality and reads containing Nextera adapters (5′-AGATGTGTATAAGAGACAG-3′) resulting from imperfect size selection. We have sequenced a total of eight libraries (Supplementary files 2A,B). The resulting pool of reads was assembled using Velvet (Zerbino, 2010) to reconstruct 6–8 kb original fragments using Velvet optimizer mode (Figure 2, Supplementary file 2A,B). Resulting contigs from Velvet were treated as input reads for downstream assembly with Celera Assembler (Myers et al., 2000) version 6.1, to produce 356a draft assembly (Figure 2—figure supplement 3, Supplementary file 2D).

### Chromosome sequencing, assignment and assembly

Embryos were isolated from a wild *B. schlosseri* colony from Monterey Bay. Metaphase chromosomes were isolated as previously described (Shoguchi et al., 2004). *B. schlosseri* metaphase chromosome suspension was partitioned into wells in the microfluidic device as previously described (Fan et al., 2011; Xu et al., 2011). The contents of each microfluidic well were amplified individually and prepared for sequencing. Each well contained between 1–4 metaphase chromosomes (Figure 3, Figure 3—figure supplement 1, Figure 3—figure Supplement 2). 21 wells were made into libraries and sequenced using Illumina Hiseq (2 x 100).

#### Chromosome assignment

Since a particular chromosome has an equal chance of occupying any of the 21 wells, we can denote the presence of a particular chromosome for example chromosome A across the 21 wells in the form of a vector (1,0,1,0,1,0,1,0,0,0,0,0,1,0,0,0,1,0,0,0,1) where 1 denotes presence in a well and 0 as absent. To deduce this configuration and perform the assignment of contigs, we aligned the reads from each well onto the contigs of the assembly. Contigs that share the same configuration across 21 wells can be considered to be from the same chromosome. To determine which contigs belong together and share the same configuration, we cluster the contigs using the Pearson correlation with each other using the vector across 21 wells. The number of clusters that result from k-means clustering using the Pearson correlation as the distance is then inferred to be the number of chromosomes. To determine this optimal number of clusters K for K-means clustering, we perform the clustering procedure iteratively for each K and record the within sum of squares error for each iteration as shown in Figure 3. Additionally, using the clustered data, we were able to deduce the configuration and deduce the number of chromosomes in each well.

Reads from each of the chromosome preparation were aligned to the 356a draft genome assembly using the BWA (Li and Durbin, 2009) package. Subsequently, SAMtools (Li et al., 2009) were used to filter for high quality mapping of reads with MAPQ score of greater than 30. The filtering was performing by using AWK to filter the fifth column of the SAM file for alignments >25. In addition to filter for a reasonable insert size, AWK is used to filter the corresponding columns in SAM files. Filtered SAM files were then parsed using BEDtools (Quinlan and Hall, 2010) to obtain the number of reads that are associated with each of the scaffolds. Specifically, the coverageBED command is used to calculate the number of reads and coverage associated with each of the scaffolds in the assembled genome. Utilizing the number of reads associated with each scaffold from a particular chromosome preparation were arranged in the following data format:

**Table:** 

Names of scaffold	Chromosome preparation 1	…	Chromosome preparation n
scaffold 1	Number of reads associated with the scaffold and preparation	…	…
…	…	…	…
Scaffold n	…	…	…

In each cell the number of reads associated with the scaffold and preparation was normalized by the total number of reads across each chromosome preparation. This is done by dividing the number of reads associated with the particular scaffold and preparation by dividing each entry by the total number of reads from a chromosome preparation. This value is then scaled by the fractional coverage of reads for a particular scaffold. These normalization steps ensure that a valid comparison can be made across each preparation. These normalized reads are then used to perform the K-means clustering. The optimal number of clusters is determined by iteratively performing the clustering process with each value of K. Within sum of square errors for each cluster is calculated and plotted in Figure 3A. A knee exists in the curve near K = 13, after which increasing the number of clusters only creates marginal improvement in the error (Figure 3A). To estimate the configuration after the clustering step, 17 out of the 21 wells were deduced to contain information that is used in the clustering process. The average number of normalized reads counts from each well that align to each scaffold in a cluster group is calculated and plotted in Figure 3B, Figure 3—figure supplements 1 and 2. Each peak represented can be inferred to denote the presence of a specific chromosome in the well. This approach yielded 13 well resolved chromosomes (Figure 3, Figure 3—figure supplement 1), close to the ∼16 chromosomes that were predicted by a previous study using metaphase spreads (Colombera, 1963).

#### 356a-chromosomes hybrid assembly

Reads from each of the individual chromosome sample preparation were subsequently assembled using Velvet (Zerbino, 2010; Figure 3—figure supplement 2). Velvet was compiled for our assembly to have a max hash length of 75. This is to allow for the use of larger hash length for the assembly of the reads from the individual well. Since the paired reads from each preparation is ∼86 after filtering for low quality bases, an optimal hash length is selected from the range of 51–75 to obtain the optimal assembly for each preparation. The assembled chromosome level contigs were then merged with 356a draft assembly using Minimus2 http://sourceforge.net/apps/mediawiki/amos/index.php?title=Minimus2 (Figure 3—figure supplement 3). During this stage, minimus2 used a nucmer based overlap detector to detect overlap between sequences and subsequently merging the two sequence sets to generate the final merge assembly of chromosome with the draft. An overall improvement in N50 was achieved, yielding a final 580 Mbp draft 356a-chromosomes hybrid assembly (Supplementary file 2D).

### Evaluation of 356a-chromosomes hybrid assembly

All of the 66 *B. schlosseri* genes, 11 fosmid sequences and most of the 98,611 expressed sequence tags (ESTs) available from NCBI aligned with the *B. schlosseri* final draft assembly (Supplementary file 2F; Figure 2—figure supplement 5). Nearly all of the independently sequenced and assembled Roche 454 Sc6a-b contigs (93%) were successfully mapped to the final assembly (Supplementary file 2F). The Sanger sequenced NCBI genes and ESTs, and the 454 and Illumina GAII sequenced Sc6a-b genome (∼11x fold coverage, Supplementary file 2C), provide validation and an independent test to the quality and integrity of the final assembly.

### RNA sequencing

RNA was isolated from 19 different individuals (developmental stages A–D; different ages). To minimize DNA contamination, colonies were starved for 48 hr before sampling. Total RNA was extracted following the manufacturer’s instructions (Ambion; Purelink RNA mini kit) and purified using the Purelink DNase kit (Invitrogen). cDNA libraries for Illumina HiSeq and MiSeq were prepared (Ovation RNA-Seq v1 system, Nugen; NEBnext DNA Master Mix for Illumina (New England Biolabs) and standard Illumina adapters and primers from IDT. RNA-Seq (2x100 bp; Illumina HiSeq) was performed. Each genotype was sequenced separately. In total, ∼88 Gb of raw transcriptome sequence data were generated for the 19 colonies (Supplementary file 2C). To investigate expression of genes in particular tissues, we also generated tissue-specific RNA-Seq libraries using the Illumina GAII (single-end 36 bp reads) and Illumina MiSeq (100-bp paired-end). For Illumina GAII RNAseq total RNA was isolated following Invitrogen’s recommendations. PolyA+ mRNA were isolated using oligoT Dynal beads. Reverse transcription and library construction protocols were provided by Illumina. In total, ∼4 Gb of raw transcriptome sequence data were generated for the tissue specific (Supplementary file 2C).

### Gene prediction

Using Cufflinks (Trapnell et al., 2010) with default parameters, *B. schlosseri* cDNA reads were aligned to the draft assembly and a reference-guided transcript assembly was produced. To predict genes, we used the program Augustus v2.5.5 (Stanke et al., 2008). The reference-guided transcripts assembly was aligned to the draft genome assembly and a ‘Hints’ gff file was generated to guide gene prediction. Augustus was run (using human HMM and parameters), and from 121,094 contigs, a total of 38,730 genes with a minimum of 30% transcript support were predicted (Supplementary file 2H).

### Gene annotation

All *B. schlosseri* candidate protein-coding genes were compared to human and mouse proteomes (UniProtKB/Swiss-Prot; see Sequence data in ‘Materials and methods ’ under ‘Phylogenomic analyses’) using blastp and an e-value threshold of e^−10^ (see Sequence Data, ‘Materials and methods’ under ‘Evolution analysis’). In addition, all *B. schlosseri* candidate protein-coding genes were compared to the NCBI non-redundant protein database (NR) using blastx and an e-value threshold of e^−10^. For every predicted gene 2 annotations were assigned (1). The best hit (smallest e-value) from NR and (2). if available, the best hit from the UniProt mouse/human blastp results.

### Evaluation of genes

RNA from *B. schlosseri* endostyles, vasculature (ampullae) and blood cells were isolated using an Ambion Purelink RNA minikit and cDNA prepared using Protoscript AMV LongAmp Taq RT-PCR kit (NEB). cDNA was amplified using GE Illustra Hot Start RTG beads and amplified using primers designed for the tested genes.

For the amplification and Sanger sequencing of the putative genes from specific blood groups, specific primers were used. Per every gene, expression was tested using cDNA prepared from endostyle, vasculature and blood. PCR was performed on the MJ Research PTC-200 thermal cycler as follows: Initial denaturation at 95°C for 4 min, followed by 34 cycles of 95°C for 1 min, 59ofor 1 min, 72°C for 1 min, followed by a final extension 72°C for 20 min. Amplified products were run on an E-Gel EX 1% agarose gel (Invitrogen) to validate size, then sent to MCLAB (384 Oyster Pt Rd. S. San Francisco, CA) for sequencing. We tested a set of 145 predicted genes by PCR and Sanger-sequencing, and were able to confirm 144 of them (99.3%), further validating the genome assembly (Figure 2—figure supplement 6).

### Mitochondrial phylogeny

#### Mitochondrial genome sequencing

Two mt-like scaffolds were initially identified in the whole-genome assembly of the *B. schlosseri* Sc6a-b specimen (Pacific Ocean, Santa Cruz harbor, California) assembled from Roche 454, Illumina GA II (Supplementary file 2D). Although these scaffolds covered the entire mitochondrial genome (mtDNA), their sequences were further validated by a conventional mtDNA sequencing strategy. Indeed, these mt scaffolds showed several ORF frameshifts and dozens of single-nucleotide deletions/insertions (indels) compared to the unpublished mtDNA of a *B. schlosseri* ‘Ve’ specimen coming from the Mediterranean Sea (Venice Lagoon, Italy). The identified frameshifts and indels were distributed almost uniformly along the mt genome sequence, and most of them fell inside or close to homopolymeric tracts longer than 7 bp, that is at the hot spots of Roche 454 sequencing errors. Thus, the complete mtDNA of the Sc6a-b specimen was amplified in two short (1.8–2.2 kb) and two long (5–7.4 kb) overlapping fragments (data available on request) using the high-fidelity Takara LA Taq enzyme (Takara) and according to the manufacturer’s instructions. Amplicons were directly sequenced using a primer walking strategy, or used as template for nested/semi-nested PCRs in order to obtain fragments easily cloneable. All mt amplicons containing several homopolymeric tracts at short distance were cloned and then sequenced, as the direct sequencing of amplicons containing homopolymer tracts always produced low-quality sequences. Cloning was carried out with the CloneJET PCR Cloning Kit (Fermentas) and sequences were obtained as the consensus of three different clones. Sanger sequencing was performed by the Eurofins MWG-Operon. The mtDNA of the *B. schlosseri* Sc6a-b specimen is 14,928 bp long and contains the typical complement of tunicate mt genes (24 tRNAs, 2 rRNAs and 13 proteins).

#### Mitochondrial phylogenetic analyses

Phylogenetic reconstructions of tunicates and deuterostomes were performed on the amino acid sequences of the 13 mitochondrially-encoded proteins. Sequences were extracted from revised entries of complete mitochondrial genomes collected in the MitoZoa database (D’Onorio de Meo et al., 2012) The analyzed taxon sample consists of 66 species, that is, all available species of Tunicata, Cephalochordata and Xenoturbellida; almost all available species of Hemichordata and Echinodermata; few representatives of Vertebrata; and two outgroup species (the arthropod *Drosophila melanogaster* and the mollusk *Aplysia californica*) (Supplementary file 1). Outgroups were selected among taxa closely related to deuterostomes, in order to ensure the analysis of the largest possible set of unambiguously aligned sites. Moreover, priority was given to outgroup species that underwent genome-sequencing projects. Among Tunicata, the larvacean *Oikopleura dioica* was not examined because of the partial status of the mtDNA and its very fast substitution rate (Denoeud et al., 2010). Few echinoderms were also excluded from the analyses due to their close relationship to other examined species. The mtDNA of the hemichordate *Rhabdopleura compacta* was excluded because of its unusual amino acid composition, causing a very long branch in phylogenetic reconstructions (Perseke et al., 2011).

The mitochondrially-encoded proteins were individually aligned with MATTF v.6 (Katoh et al., 2005), and each alignment was manually optimized. Poorly aligned positions were identified and removed by Gblocks version 0.91b (Castresana, 2000), applied with relaxed selection parameters. Individual protein alignments were then concatenated, resulting in a total alignment of 2489 amino acid sites.

Phylogenetic reconstructions were performed with Bayesian and Maximum Likelihood methods. Since the non-parametric CAT mixture model has been shown to be less prone to Long Branch Attraction (LBA) artifacts compared to other models (Lartillot et al., 2007, 2009; Philippe et al., 2009), Bayesian inferences were carried out using PhyloBayes version 3.3 (Lartillot et al., 2009) under the CAT model. In particular we applied the GTR+G+CAT model: the General Time Reversible (GTR) was used as a substitution rate matrix; the rate heterogeneity across sites was modeled according to a gamma (G) distribution with four discrete categories; and the equilibrium frequency profile was modeled with a Dirichlet Process (DP). Two independent Monte Carlo Markov chains were run for each dataset, and chain convergence was estimated checking the values of maximum discrepancy across bipartitions (<0.1) and minimum effective size of the summary variables listed in the ‘trace’ output (>100). The same tree topologies and similar posterior probabilities were obtained modeling the rate of heterogeneity with a Dirichlet process instead of a gamma distribution (GTR+DP+CAT model instead of GTR+G+CAT). The final 50% majority rule consensus tree was computed using the converged chains, discarding the initial 10% points (burn-in) and saving one point every ten cycles. The two independent chains were run for a total of 10,000 cycles, with a burn-in of 1000 cycles.

Phylogenetic trees were also inferred with PhyML 3.0 (Guindon and Gascuel, 2003), with bootstrap values based on 100 replicates. The substitution model was set to MtArt+G+F (with F = observed amino acid frequencies), that is, the model best fitting to the alignment data, as estimated by ProtTest 3 among 80 candidate models according to both the AIC and BIC selection criteria (Abascal et al., 2005).

#### *B. schlosseri* phylogenetic position

The mitochondrial phylogeny of Figure 1—figure supplement 1 shows the position of *B. schlosseri* within tunicates and deuterostomes, as reconstructed by the CAT model. This phylogeny is in perfect agreement with recent phylogenomic reconstructions based on hundreds of nuclear-encoded proteins (Bourlat et al., 2006; Delsuc et al., 2006). Indeed, the tree identifies Tunicata as a sister group of Vertebrata (thus forming the clade Olfactores) and Cephalochordata as basal to all other chordates. Moreover, it recovers with high statistical support the monophyly of Chordata and all other deuterostome phyla/subphyla (Echinodermata, Hemichordata, Cephalochordata, Vertebrata and Tunicata), as well as the clustering of Echinodermata and Hemichordata in the Ambulacraria clade.

The reconstructed phylogeny highlights extremely long branches for all tunicate species, including *B. schlosseri*, confirming that the mt evolution is characterized by a high substitution rate in all currently available tunicate sequences (see also Singh et al., 2009). This fast evolution causes a clear LBA phenomenon in the PhyML reconstruction, with the artifactual positioning of tunicates basal to all other deuterostomes (data not shown).

As for relationships within tunicates, Stolidobranchia form a monophyletic clade: *B. schlosseri* clusters with all other available Styelidae species, while the three Pyuridae species make a paraphyletic group. The paraphyly of Pyuridae (or even Styelidae) has been already observed in large molecular phylogenetic trees of Stolidobranchia based on single mitochondrial or nuclear genes, although with contrasting supports depending on the method and the selected outgroup (Zeng et al., 2006; Perez-Portela et al., 2009; Tsagkogeorga et al., 2009). Our phylogeny, based on all 13 mitochondrially-encoded proteins, strongly supports the paraphyly of Pyuridae, ad waits for additional Stolidobranchia species/families to define the monophyletic/paraphyletic status of Styelidae.

The two orders of Aplousobranchia and Phlebobranchia cluster together, supporting the morphological subdivision of ascidians into Enterogona (i.e., Phlebobranchia + Aplousobranchia) and Pleurogona based on gonad position (Garstang, 1928). Phlebobranchia also appear as a paraphyletic group since the family Cionidae clusters to Aplousobranchia, while the family Ascidiidae forms the basal branch inside the Aplousobranchia + Phlebobranchia group. Finally, the only available thaliacean species, *Doliolum nationalis*, is located basal to all Enterogona, supporting the paraphyletic nature of the class Ascidiacea. It should be noted that most published molecular phylogenies of tunicates identify a stable clade including Aplousobranchia, Phlebobranchia and Thaliacea but fail to unambiguously resolve the relationships inside this clade (see Tsagkogeorga et al., 2009 and references therein). For example, Thaliacea are alternately recovered as sister group of all Enterogona (Yokobori et al., 2006; Tsagkogeorga et al., 2009) or of the only Phlebobranchia (Swalla et al., 2000; Yokobori et al., 2005; Zeng et al., 2006). Furthermore, the sister relationship between Cionidae and Aplousobranchia has been already supported by the analysis of the single mt *cox1* gene in a large ascidian sample (Turon and Lopez-Legentil, 2004), hence confirming the inclusion of Cionidae within Aplousobranchia proposed by Kott (Kott, 1990) on the basis of morphological characters. Noteworthy, in our phylogenetic reconstruction the Aplousobranchia + Phlebobranchia + Thaliacea clade is highly supported, but the PP values of the internal nodes (i.e., the nodes suggesting the Phlebobranchia paraphyly and the basal position of *Doliolum*) are not the maximum ones (0.82 and 0.88 in Figure 1—figure supplement 1). Moreover, this portion of the tree has a different topology in the PhyML and PhyloBayes trees reconstructed using more appropriate outgroup species (i.e., only Vertebrata, Agnatha, Cephalochordata, or Agnatha+Cephalochordata; Phylobayes according to GTR+G+CAT model with 10,000 cycles) (data not shown). In particular, in these trees, *Doliolum* groups to Ascidiidae, with Cionidae forming the basal group, while Aplousobranchia is a sister group of Thaliacea + Phlebobranchia (data not shown). These observations, together with the longest branches of Aplousobranchia and the availability of a single thaliacean sequence, suggest instability of this portion of the tree as a result of long branches and insufficient taxon sampling. Thus, the relationships between Aplousobranchia, Phlebobranchia and Thaliacea need to be further investigated through the analysis of additional species.

### Phylogenomic analyses

#### Sequence data

The following well-annotated proteomes were downloaded from the UniProt Knowledgebase (UniProtKB; www.uniprot.org) on 06/29/2012, using the ‘complete proteome set’ filter: *A. queenslandica*, *B. floridae*, *C. intestinalis*, *D. melanogaster*, *D. rerio*, *G. gallus*, *H. sapiens*, *M. musculus*, *N. vectensis*, *S. purpuratus*, and *X. tropicalus*. All protein sequences associated with the *M. brevicollis* genome were downloaded from UnitProtKB on 07/07/2012. We also obtained proteomes from *A. digitifera* (v1.0.1; August 2011 update; http://marinegenomics.oist.jp/genomes/download) and *P. marinus* (assembly 7.0, release 67; known and novel gene predictions; www.ensembl.org). Finally, we included all 20,307 *B. schlosseri* predicted proteins with at least one intron and with RNA-Seq transcript support for at least 30% of the protein sequence.

#### Orthologous gene identification and alignment

An all-against-all blastp comparison was performed on the 15 proteomes listed above, with an e-value threshold of 1e−10, and otherwise default search parameters. To identify potentially orthologous genes, we analyzed the blastp output for bi-directional best hits (BBH; Kristensen et al., 2011), defined as pairs of mutually best-matching protein sequences from different species. We reduced the impact of confounding paralogs by employing a simple filter, as described previously (Srivastava et al., 2010). As a result, 521 protein networks (or clusters) were identified, each of which covers at least 12 of the 15 analyzed proteomes. To assemble a sequence matrix for phylogenetic inference, each protein cluster was aligned using ClustalW (Larkin et al., 2007) (default parameters), trimmed for high-quality alignment blocks with GBlocks (Castresana, 2000) (default settings), sorted by species name, and concatenated. Strings of ‘X’ characters were introduced into the alignment for species with missing proteins. The final alignment matrix consisted of 15 species by 40,798 amino acids (521 nuclear genes).

#### Maximum likelihood and Bayesian analyses

The evolutionary position of *B. schlosseri* was compared among the 15 species in the alignment matrix (above) using the PhyML 3.0 software tool (Guindon and Gascuel, 2003) (with default parameters), which implements a maximum likelihood approach for phylogenetic tree reconstruction. The resulting tree is illustrated in Figure 1E.

As an alternative approach, we employed PhyloBayes3.3c, a non-parametric method incorporating Bayesian statistics and Markov Chain Monte Carlo sampling (Lartillot et al., 2009). Importantly, PhyloBayes accounts for site-specific evolutionary effects, and by using non-parametric methods, can learn the distribution of each site’s amino acid profile and evolutionary rate directly from the data. Using a general time reversible (GTR) process to model exchange rates, and otherwise default parameters (CAT model: Dirichlet process of equilibrium frequency profiles, in which all parameters are learned from the data; branch lengths ∼ iid gamma), we ran two MCMC chains in parallel. After 1971 cycles, both chains were convergent. We subsequently generated majority-rule consensus trees for each chain by discarding the first 100 trees (burn-in), followed by sampling every other 10 trees. We required a posterior probability threshold of 0.95 for the final consensus tree, as only nodes achieving this threshold are considered well supported (Lartillot et al., 2009). The final consensus tree was topologically consistent with the maximum likelihood tree (Figure 1E), supporting the inferred evolutionary position of *B. schlosseri* among the analyzed proteomes.

### Evolution analysis

#### Identification of *B. schlosseri* genes potentially linked to the emergence of vertebrate phenotypes

To gain insights into individual genes underlying the emergence of vertebrates, we analyzed 19 proteomes for the presence or absence of genes displaying at least some homology to human/mouse proteomes. In addition to the 15 proteomes used for the phylogenomic analysis, we used the following well-annotated proteomes from UniProtKB (*C. elegans* and *Oikopleura dioica*, downloaded 06/29/2012; *Arabidopsis thaliana* and *Neurospora crassa*, downloaded 07/07/2012). Moreover, for completeness, we included all predicted *B. schlosseri* protein-encoding genes, including gene fragments. After running all-vs-all blastp, and applying an e-value cutoff of e^−10^, a binary matrix was assembled depicting the presence or absence of each gene as 1 or 0, respectively (Figure 4—source data 1A).

#### Evolution of hematopoiesis and identification of candidate hematopoietic precursor genes in *B. schlosseri*

To identify candidate evolutionary precursors of human blood cell populations in *B. schlosseri*, we examined the evolutionary progression of gene sets with expression patterns restricted to distinct immune cell populations. These gene sets were defined by re-analysis of data from a recent publication (Benita et al., 2010), in which tissue/cell type-specific enrichment scores were derived for the expression of each gene in the human transcriptome, across 126 human tissues spanning 557 arrays profiled on the same microarray platform (Affymetrix U133A) (http://xavierlab2.mgh.harvard.edu/EnrichmentProfiler/). We analyzed the top 20 most enriched genes for each of 26 representative immune cell populations (Supplementary file 3), and interrogated a broad taxonomic range of proteomes for the presence or absence of each gene (using the binary matrix in Figure 4—source data 1A). Importantly, we confirmed cell-type restricted expression patterns for representative gene sets using a database of highly purified mouse hematopoietic populations that we recently developed (Seita et al., 2012). A blastp e-value threshold of e^−10^ was used to establish minimal acceptable homology to human/mouse proteomes. Results are rendered as a heat map in Figure 5.

### Data deposition

The sequence of the *B. schlosseri* mitochondrial genome has been submitted to the European Nucleotide Archive under accession number HF548551.

An integrated genome and transcriptome browser of *B. schlosseri* has been developed and is available at: http://genepyramid.stanford.edu/botryllusgenome/.

## Additional files

Supplementary file 1. Species classification and Accession Number (AC) of the complete mtDNA sequences used in the tunicate phylogenetic reconstructions. **DOI:** http://dx.doi.org/10.7554/eLife.00569.021

Supplementary file 2. *B. schlosseri* genome statistics. (**A**) Statistics of Velvet assembly of wells per every 356a genomic library preparation. (**B**) Detailed statistics of Velvet assembly of wells per every 356a genomic library preparation. Name: barcode number; #reads: number of filtered reads obtained, NumCtgs: number of assembled contigs, Min: minimum contig length obtained, Max: maximum contig length obtained, Middle: length of the middle contig; Sum: sum of assembled contigs (bp), N50: length of N50 (bp), NumAbove8k: number of assembled contigs above 8k, NumAbove20k: number of assembled contigs above 20k. Kmer: Kmer used by Velvet assembly, Ecov: estimated coverage, CovCutoff: minimum coverage used. (**C**) Total gDNA and RNA sequence data obtained. Genomic DNA was extracted from tissue from two long-lived colonies (356a and Sc6a-b) raised in our mariculture facility. Sequence reads from colony Sc6a-b were obtained using Roche 454 Titanium and Illumina GAIIx. Sequence reads from colony 356a and 21 individual chromosomes isolated from a wild colony were obtained by Illumina HiSeq 2000. RNA-seq reads from several tissues (endostyle, vasculature, gonads and digestive system) were obtained by Illumina GAIIx and Illumina MiSeq. RNA-seq reads from 19 different lab reared colonies were obtained by Illumina HiSeq 2000. (**D**) *B. schlosseri* genome assemblies and chromosome scaffolds statistics. (**E**) Interspersed repeats in *B. schlosseri.* Analysis of interspersed repeat content in *B. schlosseri*, compared to *C. intestinalis* (version ci2, downloaded from the UCSC genome browser). RepeatScout (version 1.0.5; Price et al., 2005) was used to identify interspersed repeat elements de novo using a k-mer length of 14. All identified repeats were subsequently filtered for tandem repeat and low complexity content, using RepeatScout. Genome-wide interspersed repeats were catalogued using RepeatMasker (version open-4.0) (Smit et al., 1996–2010). (**F**) Alignment statistic of all of the *B. schlosseri* non mitochondrial genes (66), expressed sequence tags (98,611 EST’s) and fosmids (11) available on NCBI and, Sc6a-b assembly (518,856 contigs) with the *B. schlosseri* 356a chromosome hybrid draft genome assembly. (**G**) Genome assemblies statistics of several wild type species. (**H**) *B. schlosseri* predicted gene structure statistics. (**I**) Barcoded adapters list for 192 wells. **DOI:** http://dx.doi.org/10.7554/eLife.00569.022

Supplementary file 3. Potential precursors of human hematopoietic populations in *B. schlosseri*. We analyzed gene expression microarray data from 26 different human blood cell populations, along with a large diversity of non-blood human tissue samples, and identified a set of twenty signature genes with highly enriched expression profiles for each hematopoietic population. The 20 signature genes for each blood-related gene set, and the identified orthologous gene sequences in *B. schlosseri* are presented. Status = 1 predicted gene is present in *B. schlosseri* genome; Status = 0 predicted gene is absent from *B. schlosseri* genome. **DOI:** http://dx.doi.org/10.7554/eLife.00569.023

## Major datasets

The following datasets were generated:

VoskoboynikA, NeffNF, SahooD, NewmanAM, PushkarevD, KohW, PassarelliB, FanCH, MantalasGL, PalmeriKJ, IshizukaKJ, GissiC, GriggioF, Ben-ShlomoR, CoreyDM, PenlandL, WhiteRAIII, WeissmanIL, QuakeSR, 2013, The genome sequence of the colonial chordate, Botryllus schlosseri, ; http://www.ncbi.nlm.nih.gov/bioproject/PRJNA205369, Publicly available at NCBI BioProject (http://www.ncbi.nlm.nih.gov/bioproject).

VoskoboynikA, NeffNF, SahooD, NewmanAM, PushkarevD, KohW, PassarelliB, FanCH, MantalasGL, PalmeriKJ, IshizukaKJ, GissiC, GriggioF, Ben-ShlomoR, CoreyDM, PenlandL, WhiteRAIII, WeissmanIL, QuakeSR, 2013, Botryllus schlosseri complete mitochondrial genome, isolate sc6a-b, ; http://www.ebi.ac.uk/cgi-bin/sva/sva.pl?search=Go&query=HF548551, Publicly available at the European Nucleotide Archive (http://www.ebi.ac.uk/ena/).

VoskoboynikA, NeffNF, SahooD, NewmanAM, PushkarevD, KohW, PassarelliB, FanCH, MantalasGL, PalmeriKJ, IshizukaKJ, GissiC, GriggioF, Ben-ShlomoR, CoreyDM, PenlandL, WhiteRAIII, WeissmanIL, QuakeSR, 2013, Botryllus schlosseri Genome Project, http://genepyramid.stanford.edu/botryllusgenome/, Publicly available

The following previously published datasets were used:

BhatlaM, 2006, Molecular signatures orchestrating the fate of human hematopoietic stem cells originating from different stages of ontogeny, ; http://www.ncbi.nlm.nih.gov/geo/query/acc.cgi?acc=GSE3823, Publicly available at the Gene Expression Omnibus (http://www.ncbi.nlm.nih.gov/geo/).

EckfeldtCE, MendenhallEM, FlynnCM, WangT, GrindleSM, EkkerSC, VerfallieCM, PickartMA, 2005, Human Hematopoietic Stem Cell (HSC) and Progenitor Cell (HPC) expression profilies, ; http://www.ncbi.nlm.nih.gov/geo/query/acc.cgi?acc=GSE2666, Publicly available at the Gene Expression Omnibus (http://www.ncbi.nlm.nih.gov/geo/).

GustafssonC, MjüsbergJ, MatussekA, GeffersR, MatthiesenL, BergG, SharmaS, BuerJ, ErnerudhJ, 2008, Gene expression profiling of human decidual macrophages: evidence for immunosuppressive phenotype, ; http://www.ncbi.nlm.nih.gov/geo/query/acc.cgi?acc=GSE10612, Publicly available at the Gene Expression Omnibus (http://www.ncbi.nlm.nih.gov/geo/).

HutchesonJ, ScatizziJC, SiddiquiAM, HainesGK3rd, WuT, LiQZ, DavisLS, MohanC, PerlmanH, 2008, Combined deficiency of proapoptotic regulators Bim and Fas results in the early onset of systemic autoimmunity, ; http://www.ncbi.nlm.nih.gov/geo/query/acc.cgi?acc=GSE10325, Publicly available at the Gene Expression Omnibus (http://www.ncbi.nlm.nih.gov/geo/).

HyrczaMD, KovacsC, LoutfyM, HalpennyR, HeislerL, YangS, WilkinsO, OstrowskiM, DerSD, 2007, Distinct transcriptional profiles in ex vivo CD4+ and CD8+ T cells are established early in human immunodeficiency virus type 1 infection and are characterized by a chronic interferon response as well as extensive transcriptional changes in CD8+ T cells, ; http://www.ncbi.nlm.nih.gov/geo/query/acc.cgi?acc=GSE6740, Publicly available at the Gene Expression Omnibus (http://www.ncbi.nlm.nih.gov/geo/).

JuraJ, WegrzynP, KorostyńskiM, GuzikK, Oczko-WojciechowskaM, JarzabM, KowalskaM, PiechotaM, PrzewłockiR, KojA, 2007, Identification of interleukin-1 and interleukin-6-responsive genes in human monocyte-derived macrophages using microarrays, ; http://www.ncbi.nlm.nih.gov/geo/query/acc.cgi?acc=GSE8515, Publicly available at the Gene Expression Omnibus (http://www.ncbi.nlm.nih.gov/geo/).

LeeMS, HanspersK, BarkerCS, KornAP, McCuneJM, 2004, Gene expression profiles during human CD4+ T cell differentiation, ; http://www.ncbi.nlm.nih.gov/geo/query/acc.cgi?acc=GSE1460, Publicly available at the Gene Expression Omnibus (http://www.ncbi.nlm.nih.gov/geo/).

LiuH, ShiB, HuangCC, EksarkoP, PopeRM, 2007, Transcriptional diversity during monocyte to macrophage differentiation, ; http://www.ncbi.nlm.nih.gov/geo/query/acc.cgi?acc=GSE8286, Publicly available at the Gene Expression Omnibus (http://www.ncbi.nlm.nih.gov/geo/).

LundRJ, AittokallioT, NevalainenO, LahesmaaR, KainonenE, LahesmaaA, DixonC, AhlforsH, MelinO, MillerM, 2005, Identification of novel genes regulated by IL-12, IL-4, or TGF-beta during the early polarization of CD4+ lymphocytes, ; http://www.ncbi.nlm.nih.gov/geo/query/acc.cgi?acc=GSE2770, Publicly available at the Gene Expression Omnibus (http://www.ncbi.nlm.nih.gov/geo/).

MartensJH, KzhyshkowskaJ, Falkowski-HansenM, SchledzewskiK, GratchevA, MansmannU, SchmuttermaierC, DippelE, KoenenW, RiedelF, GoerdtS, 2006, Differential expression of a gene signature for scavenger/lectin receptors by endothelial cells and macrophages in human lymph node sinuses, the primary sites of regional metastasis, ; http://www.ncbi.nlm.nih.gov/geo/query/acc.cgi?acc=GSE2665, Publicly available at the Gene Expression Omnibus (http://www.ncbi.nlm.nih.gov/geo/).

OcklenburgF, Moharregh-KhiabaniD, GeffersR, JankeV, PfoertnerS, GarritsenH, GroebeL, KlempnauerJ, DittmarKE, WeissS, BuerJ, Probst-KepperM, 2006, UBD, a downstream element of FOXP3, allows the identification of LGALS3, a new marker of human regulatory T cells, ; http://www.ncbi.nlm.nih.gov/geo/query/acc.cgi?acc=GSE4527, Publicly available at the Gene Expression Omnibus (http://www.ncbi.nlm.nih.gov/geo/).

PopovA, DriesenJ, AbdullahZ, WickenhauserC, BeyerM, Debey-PascherS, SaricT, KummerS, TakikawaO, DomannE, ChakrabortyT, KrünkeM, UtermühlenO, SchultzeJL, 2008, Infection of myeloid dendritic cells with Listeria monocytogenes leads to the suppression of T cell function by multiple inhibitory mechanisms, ; http://www.ncbi.nlm.nih.gov/geo/query/acc.cgi?acc=GSE9946, Publicly available at the Gene Expression Omnibus (http://www.ncbi.nlm.nih.gov/geo/).

SeitaJ, SahooD, RossiDJ, BhattacharyaD, SerwoldT, InlayM, EhrlichLI, FathmanJW, DillDL, WeissmanIL, 2012, Gene expression common: an open platform for absolute gene expression profiling, ; http://www.ncbi.nlm.nih.gov/geo/query/acc.cgi?acc=GSE34723, Publicly available at the Gene Expression Omnibus (http://www.ncbi.nlm.nih.gov/geo/).

D’Onorio de MeoP, D’AntonioM, GriggioF, LupiR, BorsaniM, PavesiG, CastrignanüT, PesoleG, GissiC, 2012, MitoZoa 2.0: a database resource and search tools for comparative and evolutionary analyses of mitochondrial genomes in Metazoa, ; http://mi.caspur.it/mitozoa/
